# Synthesis and Antifungal Activity against *Fusarium oxysporum* of Some Brassinin Analogs Derived from l-tryptophan: A DFT/B3LYP Study on the Reaction Mechanism

**DOI:** 10.3390/molecules21101349

**Published:** 2016-10-11

**Authors:** Diego Quiroga, Lili Dahiana Becerra, John Sadat-Bernal, Nathalia Vargas, Ericsson Coy-Barrera

**Affiliations:** Bioorganic Chemistry Laboratory, Facultad de Ciencias Básicas y Aplicadas, Universidad Militar Nueva Granada, Campus Nueva Granada, Cajicá 49300, Colombia; ldbecerrag@unal.edu.co (L.D.B.); john.bernal@unimilitar.edu.co (J.S.-B.); u0500887@unimilitar.edu.co (N.V.); ericsson.coy@unimilitar.edu.co (E.C.-B.)

**Keywords:** Brassinin, phytoalexin, l-tryptophan derivatives, dithiocarbamate, *Fusarium oxysporum*

## Abstract

An efficient methodology to obtain novel antifungal analogs of brassinin **1** is described. Starting from l-tryptophan **2**, *N*,*N′*-dialkylthiourea **4**, 4-[(1*H*-indol-3-yl)methylene]-2-sulfanylidene-1,3-thiazolidin-5-one **5** and alkyl (2*S*)-3-(1*H*-indol-3-yl)-2-{[(alkylsulfanyl)carbonothioyl]amino}propanoate **6** type compounds were obtained as main products in different ratios depending on the reaction conditions via a tandem dithiocarbamate formation and Michael addition reaction. In order to understand the dependence of the reaction conditions on the mechanism pathway, a DFT/B3LYP study was performed. The results suggested the existence of competitive mechanistic routes which involve the presence of an ionic dithiocarbamate intermediate **9**. Antifungal activities of all products were then evaluated against *Fusarium oxysporum* through mycelial growth inhibition using a microscale amended-medium assay. IC_50_ values were thus determined for each compound. These results showed that **6**-related compounds can be considered as promissory antifungal agents.

## 1. Introduction

**Figure 1:**
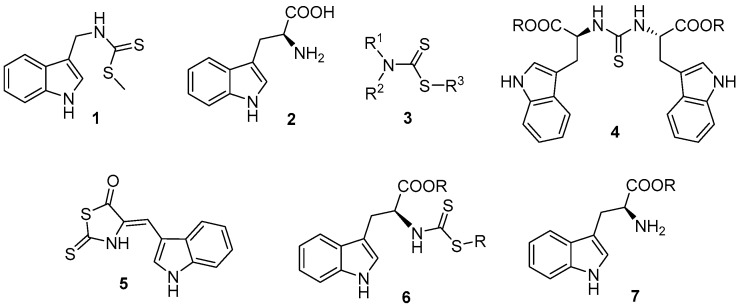
Chemical structures for compounds **1**–**7**.

Phytoalexins are secondary metabolites produced by plants for protecting against microbial pathogens, heavy metals, and UV irradiation. They are crucial components of the defense mechanisms in plants. Among the cruciferous (*Brassicaceae*) phytoalexins, brassinin **1** has a fundamental role in the biosynthetic pathway of phytoalexins derived from l-tryptophan **2**. Brassinin **1** is an important precursor of several phytoalexins such as cyclobrassinin, brassilexin, spirobrassinin, dioxybrassinin, brassicanate A, and rutalexin produced by *Brassica* spp*.* and other cruciferous species [1,2,3,4,5,6,7]. Although these plants can produce mixtures of phytoalexins having different biological effects on several pathogens, some phytopathogenic fungi transform indole phytoalexins in biologically inactive products [1,2,3,4,5,6,7]. Paldoxins, phytoalexin detoxification inhibitors, are an environmentally friendly alternative via inhibiting specific metabolic reactions in fungal phytopathogens. Some of the synthetic strategies based on heterocyclic systems, such as indole-3-substituted derivatives, have been proposed as promissory paldoxins (Figure 1) [2]. In this context, the importance of sulfur functionalized organic groups (i.e., dithiocarbamate moieties) and the presence of indole-based skeleton in this kind of compounds have been evidenced [8].

To obtain these compounds, a suitable synthetic strategy involves the reaction between amines and carbon disulfide [9,10,11]. Wang et al. [12] studied the reaction between *o*-phenylenediamine and carbon disulfide to obtain 2-mercaptobenzimidazole, using triethylamine as catalyst in THF, observing the formation of an ionic dithiocarbamate-type intermediate [12]. Therefore, the reaction between dithiocarbamic acid salts with Michael acceptors offers an ecofriendly alternative to obtaining alkyl dithiocarbamates via the formation of the above-mentioned ionic intermediate. Bardajee et al. [13] achieved the synthesis of alkyl dithiocarbamates using a solvent-free multicomponent reaction between alkylamines, carbon disulfide, and Michael acceptors in the presence of the KF and aluminum oxide mixture. Azizi et al. [14] reported a method for the synthesis of alkyl dithiocarbamates using mild reaction conditions starting from similar synthetic precursors.

Recently, our research has been directed towards the synthesis of Brassinin(**1**)-like analogs using conventional α-aminoacids as synthetic precursors to generate antifungal agents against *Fusarium oxysporum*. Here, we describe the functionalization of l-tryptophan **2** towards *N*,*N*′-dialkylthiourea **4**, 4-[(1*H*-indol-3-yl)methylene]-2-sulfanylidene-1,3-thiazolidin-5-one **5** and alkyl (2*S*)-3-(1*H*-indol-3-yl)-2-{[(alkylsulfanyl)carbonothioyl]amino}propanoate **6** type compounds. In order to understand the influence of the reaction conditions in the ratios of the mentioned products, a DFT study at the B3LYP/6-31G(d,p) level of theory was then performed. An in vitro assay against *Fusarium oxysporum* was performed to evaluate the antifungal activity of the synthesized products*.*

## 2. Results and Discussion

**Scheme 1:**
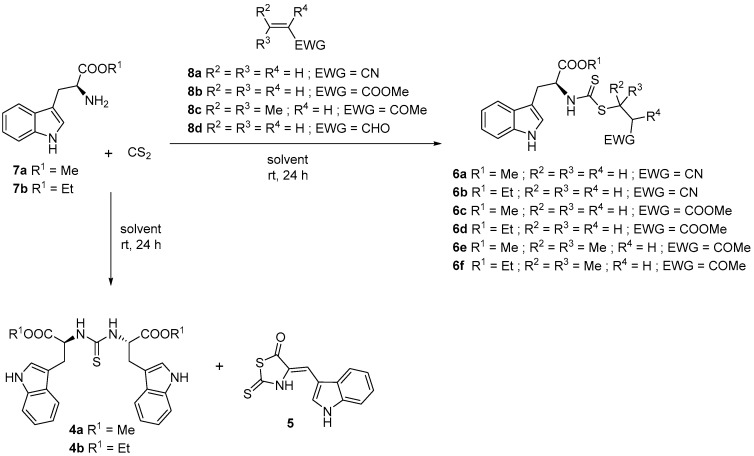
Synthesis of compounds **4**, **5**, and **6**.

The first step of the present synthetic methodology involved the esterification of **2**, as previously reported by Li and Sha [15]. To evaluate the esterification method, the reactions were performed using four different aliphatic alcohols (R = Me, Et, 2-Pr, and *n*-Bu). We synthesized alkyl (2*S*)-2-amino-3-(1*H*-indol-3-yl)propanoate compounds **7a**–**d** with yields of 85%–96%. Reactions between **7a**–**b**, carbon disulfide, and Michael acceptors **8a**–**d** were studied under different conditions such as acid-basic medium character, temperature and solvents (Scheme 1).

**Scheme 2:**
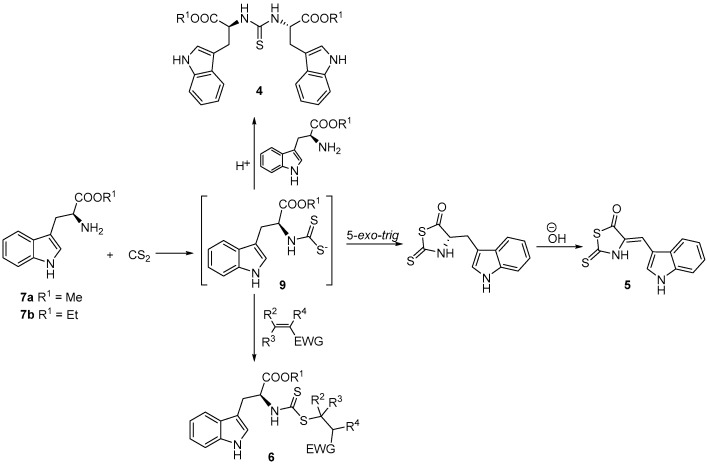
Proposed mechanism for forming **4**, **5**, and **6**.

The reaction mixtures were maintained for 24 h in constant stirring carrying out each trial at different temperatures (−10, 0, 10, and 40 °C). We identified **4a**–**b** as main products in soft acid catalysis and acetonitrile , while **5** was obtained in a soft basic medium and methanol. The formation of **6a**–**f** was detected in low yields (<5%) in both acid and basic catalysis. When reactions were carried out in strong acid or basic media at 40 °C, the number of detectable products increased. These results suggest that reaction pathways were dependent on the solvent and the medium‘s acid-basic character, implying competitive mechanistic routes. The first route involves the formation of **4** through condensation of the ionic dithiocarbamate intermediate **9** and a second molecule of **7**, **a** consecutive addition and elimination releasing sulfide hydrogen. The second route involves the 5-*exo*-*trig* intramolecular cyclization of **9** to produce **5** favored by the basic medium within a kinetic control. A third route involves the reaction of intermediate **9** with a Michael acceptor, which is not favored by the solvation effect of polar solvents (Scheme 2).

**Figure 2:**
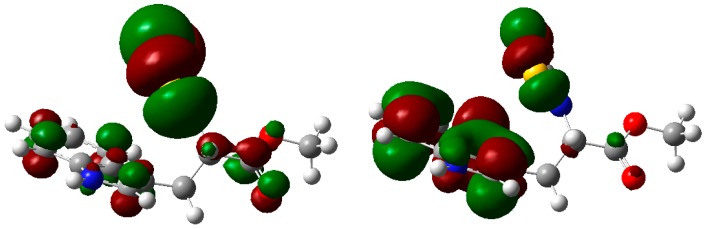
HOMO and LUMO for intermediate **9** (Isovalue of 0.02). White balls represent hydrogen atoms, gray balls represent carbon atoms, blue balls represent nitrogen atoms, red balls represents oxygen atoms, yellow balls represent sulfur atoms, dark red and green regions represent HOMO-LUMO orbitals.

Computational calculations were carried out using the DFT method at the B3LYP/6-31G(d,p) level of theory [16]. The formation of **9** is proposed as the first step in the mechanistic route. Using Fukui index values, we established both electrophilic and nucleophilic characters of the carbonyl carbon (C=O, *f*+ between 0.050 and 0.100) and the sulfur atoms (R-(C=S)-S^−^, *f*− between 0.110 and 0.400) in **9**, respectively. These results suggest strong intramolecular both electrophilic and nucleophilic characters and the intramolecular cyclization of **9** towards **5** (ΔG^0^ calculated = −5.77 kcal/mol) in a basic medium. The HOMO in **9** is located in *sp*^3^ and non-bonding orbitals in the sulfide fragment, while LUMO is partially located in the π* orbital of the C=O bond in the ester group, the π* orbital of the C=S, and the π* orbital of the aromatic rings (Figure 2). In the second route, **9** undergoes nucleophilic attack by **7** on the carbon atom of the thiocarbonyl moiety to yield hydrogen sulfide and **9** (ΔG^0^ calculated = +33.80 kcal/mol) in an acid medium. The solvation effect of intermediate **9** was evaluated using IEFPCM and IPCM models. The results suggest that the interaction between **9** and methanol tends to reduce its stability, which can be verified according to the total energy of **9** in each model (there is an energy difference of +56.8 kcal/mol and +72.9 kcal/mol, respectively). Moreover, the methanol solvation effect tends to enhance the polarizability of the C=O bond in the ester fragment according to the Mulliken charge values of the carbon atom (+0.189 in gas phase, +0.200 for each solvation model). These results support the hypothesis that the presence of a polar protic solvent in the reaction medium exerts an influence in the reaction pathway.

In order to enhance the formation of compounds **6**, some one-pot reactions of l-tryptophan **2** with carbon disulphide, Michael acceptors, trimethylsilane chloride, and the respective alcohol were carried out in dry THF employing triethylamine (TEA) as base. TMSCl and the alcohol were used to protect the carboxylic acid group in situ. The tandem dithiocarbamate formation and Michael addition reaction produced compounds **6** in higher yields (75%–95%). However, using acrolein as a Michael acceptor yielded only polymeric resins, which were therefore not characterized. The Michael addition occurred as a competitive step (Scheme 2), where intermediate **9** suffers a weak solvent effect, which lead to its stabilization as we proposed for polar solvents. Therefore, intermediate **9** attacks the electrophilic carbon atom in the Michael acceptor promoting the C–S coupling with **6**. These results demonstrate that the third route is preferred using a one-pot reaction and mild conditions.

In vitro antifungal activity testing against *Fusarium oxysporum* was performed using a microscale amended-medium assay (resistant strain G1 obtained from Cape gooseberry, provided by the collection of Phytopathology Laboratory at UMNG). Compounds **4**, **5**, **6**, and **7** were evaluated at five different concentrations in the 10–300 µg/mL range. The results were expressed as half-maximal inhibitory concentration (IC_50_ in mM) for each compound, using a non-linear regression in the program GraphPad Prism version 5.00 for Windows. All the results are summarized in Table 1.

Results for compounds **7a**–**d** suggest that the size of the substituent has an effect on antifungal activity. However, these results are unsatisfactory considering that **7a**–**d** are indole derivatives. Thus, protection of the carboxylic group does not enhance the antifungal activity of these compounds. Results for **6** showed lower IC_50_ values to that of their precursors, suggesting that the addition of a dithiocarbamate group increases the inhibitory effect on the fungus. The effect of the alkyl substituent at the ester group and the electron withdrawing group at the dithiocarbamate fragment were evident in this kind of compound. These structural moieties tend to enhance the antifungal activity, specifically using ethyl and nitrile groups. However, compounds **6e** and **6f** showed the opposite. This behavior may indicate that bulky substituents in the dithiocarbamate fragment can promote the formation of hydrophobic interactions avoiding the correct binding within specific fungal targets.

## 3. Materials and Methods 

### 3.1. General Information 

All chemicals were purchased from Sigma-Aldrich (Saint Louis, MO, USA) and Merck KGaA (Darmstadt, Germany) and used without further purification. Dry solvents were purchased in sufficient purity. Thin layer chromatography (TLC) was done on TLC silica gel 60 F_254_ (Merck KGaA), and compounds were detected at 254 nm. Column chromatography was conducted manually on silica gel 60 (0.040–0.063 mm) from Merck KGaA. Nuclear magnetic resonance (NMR) spectra were recorded on a Bruker Avance AV-400 MHz spectrometer (Billerica, MA, USA). All shifts are given below in δ (ppm) using the signal of tetramethylsilane (TMS) as a reference. All coupling constants (*J*) are given in Hz. Splitting patterns are typically described as follows: s: singlet, d: doublet, t: triplet, and m: multiplet.

The HPLC analyses were performed on a Shimadzu Prominence HPLC instrument (Kyoto, Japan) using a Synergi HydroRP-C18 column (150 mm × 4.6 mm × 4 µm) (Torrance, CA, USA). The mobile phases consisting of A (formic acid 1% in acetonitrile) and B (formic acid 0.1% in water) were used with the gradient mode at a flow rate of 1.5 mL/min. The UV detection at 270 nm was performed with a diode array detector (Shimadzu, Kyoto, Japan). The mass spectrometry experiments were performed on a LC/MS 2020 spectrometer (Shimadzu) with electrospray ionization in positive ion mode. The sign of the optical specific rotations for all the compounds was determined with a Jasco P-2000 Polarimeter (JASCO Co., Ltd., Mary’s Court, PA, USA) in a quartz cell (1.0 cm), and the value is an average of ten measures.

### 3.2. General Procedure for the Synthesis of Alkyl Esters (***7a**–**d***)

Compounds **7a**–**d** were prepared as described in the literature [15] with some modifications: trimethylsilane chloride (2 mmol) was added to a solution of l-tryptophan **2** (1 mmol) in the respective alcohol (5.0 mL). The reaction mixture was stirred at room temperature for 24 h, and the solvent was slowly evaporated at room temperature over a period of about 1 week, and the solid residue was recrystallized from ethanol. The crude reaction product in its hydrochloride form was treated with a 10% aqueous solution of sodium bicarbonate and extracted with ethyl acetate (5 × 10 mL) to yield compounds **7a**–**d**.

*Methyl (2S)-2-amino-3-(1H-indol-3-yl)propanoate* (**7a**): ESI-MS in positive mode *m*/*z*: [M − H]^+^: 219.00; [α]${}_{D}^{25}$ = + 5.80 ± 0.200 (*c* 0.1, H_2_O).

*Ethyl (2S)-2-amino-3-(1H-indol-3-yl)propanoate* (**7b**): ESI-MS in positive mode *m*/*z*: [M − H]^+^: 233.05; [α]${}_{D}^{25}$ = −7.73 ± 0.306 (*c* 0.1, H_2_O).

*2-Propyl-(2S)-2-amino-3-(1H-indol-3-yl)propanoate* (**7c**): ESI-MS in positive mode *m*/*z*: [M − H]^+^: 247.00; [α]${}_{D}^{25}$ = −21.5 ± 0.306 (*c* 0.1, H_2_O).

*n-Butyl-(2S)-2-amino-3-(1H-indol-3-yl)propanoate* (**7d**): ESI-MS in positive mode *m*/*z*: [M − H]^+^: 261.00; [α]${}_{D}^{25}$ = −15.1 ± 0.231 (*c* 0.1, H_2_O).

### 3.3. General Procedure for the Synthesis of N,N′-Dialkylthioureas (***4a**–**b***)

A 10% aqueous solution of hydrochloric acid (1.0 mL) and carbon disulfide (2 mmol) was added to a solution of the respective ester (**7a**–**d**, 1.0 mmol) in acetonitrile. The reaction mixture was stirred at room temperature for 24 h, the reaction was then neutralized with 10% aqueous solution of sodium bicarbonate, and the product was extracted with CHCl_3_ (4 × 5 mL). The extract was concentrated under reduced pressure and the residue was purified via column chromatography on Silica gel and eluted with a hexane–ethyl acetate (7:3) mixture, to afford compounds **4a**–**b**.

*Dimethyl (2S,2′S)-2,2′-(carbonothioyldiazanediyl)-3,3′-bis(1H-indol-3-yl)dipropanoate* (**4a**): Yellow oil. ESI-MS in positive mode *m*/*z*: [M − H]^+^: 479.05. 

*Diethyl (2S,2′S)-2,2′-(carbonothioyldiazanediyl)-3,3′-bis(1H-indol-3-yl)dipropanoate* (**4b**): Yellow oil. ESI-MS in positive mode *m*/*z*: [M − H]^+^: 507.05.

### 3.4. Synthesis of 4-[(1H-Indol-3-yl)methylene]-2-sulfanylidene-1,3-thiazolidin-5-one (***5***)

A 10% aqueous solution of potassium hydroxide (1.0 mL) and carbon disulfide (2 mmol) was added to a solution of the respective ester (**7a**–**d**, 1.0 mmol) in methanol. The reaction mixture was stirred at room temperature for 24 h. The product was extracted with ethyl acetate (4 × 5 mL). The extract was concentrated under reduced pressure and the residue was purified via column chromatography on Silica gel and eluted with a hexane-ethyl acetate (7:3) mixture to afford compound **5**: ESI-MS in positive mode *m*/*z*: [M − H]^+^: 261.15; [α]${}_{D}^{25}$ = −2.60 ± 0.200 (*c* 0.1, MeOH).

### 3.5. General Procedure for the Synthesis of Alkyl (2S)-3-(1H-indol-3-yl)-2-{[(alkylsulfanyl)carbonothioyl]amino}propanoate (***6a**–**f***)

Method A: Triethylamine (2 mmol), carbon disulfide (1 mmol) and the respective Michael acceptor **8a**–**d** were added to a solution of the respective ester (**7a**–**b**, 1 mmol) in THF. The reaction mixture was stirred at room temperature for 24 h. It was then concentrated under reduced pressure and the residue was purified via column chromatography on Silica gel and eluted with a hexane-ethyl acetate (7:3) mixture to yield compounds **6a**–**f**.

Method B via tandem dithiocarbamate formation/Michael addition reaction: The respective alcohol (R = Me, Et; 1 mmol), trimethylsilane chloride and triethylamine (2 mmol) were added to a solution of l-tryptophan **2** (1 mmol) in THF. Then, a solution of carbon disulfide (1 mmol) and the respective Michael acceptor **8a**–**d** in THF was added dropwise. The reaction mixture was stirred at room temperature for 24 h and it was then concentrated under reduced pressure and the residue was purified via column chromatography on Silica gel and eluted with a hexane–ethyl acetate (7:3) mixture to yield compounds **6a**–**f**.

*Methyl 2-({[(2-cyanoethyl)sulfanyl]carbonothioyl}amino)-3-(1H-indol-3-yl)propanoate* (**6a**): ^1^H-NMR (400.1 MHz, CDCl_3_): 8.20 (s, 1H), 7.26–7.13 (m, 4H), 6.84 (s, 1H), 5.56–5.74 (m, 1H), 3.80 (s, 3H), 3.47–3.66 (m, 1H), 3.09–3.20 (m, 1H), 2.9–83.05 (m, 2H), 2.87–2.98 (m, 2H). ESI-MS in positive mode *m*/*z*: [M − H]^+^: 347.80. [α]${}_{D}^{25}$ = +7.00 ± 0.200 (*c* 0.1, MeOH). 

*Ethyl 2-({[(2-cyanoethyl)sulfanyl]carbonothioyl}amino)-3-(1H-indol-3-yl)propanoate* (**6b**): ^1^H-NMR (400.1 MHz, CDCl_3_): 8.22 (s, 1H), 7.26–7.13 (m, 4H), 6.84 (s, 1H), 5.54–5.75 (m, 1H), 4.20 (q, 2H), 3.47–3.66 (m, 1H), 3.10–3.20 (m, 1H), 2.98–3.05 (m, 2H), 2.89–3.00 (m, 2H), 1.40 (t, 3H). ESI-MS in positive mode *m*/*z*: [M − H]^+^: 362.15. [α]${}_{D}^{25}$ = +4.33 ± 0.306 (*c* 0.1, MeOH).

*Methyl 3-(1H-indol-3-yl)-2-({[(3-methoxy-3-oxopropyl)sulfanyl]carbonothioyl}amino)propanoate* (**6c**): 1H-NMR (400.1 MHz, CDCl_3_): 8.15 (s, 1H), 7.55–7.10 (m, 4H), 6.97 (s, 1H), 5.49–5.56 (m, 1H), 3.60 (s, 3H), 3.58 (s, 3H), 3.51–3.55 (m, 1H), 3.46–3.50 (m, 2H), 3.38–3.44 (m, 1H), 2.65–2.87 (m, 2H). ESI-MS in positive mode *m*/*z*: [M − H]^+^: 380.80. [α]${}_{D}^{25}$ = −2.80 ± 0.917 (*c* 0.1, MeOH). 

*Ethyl 3-(1H-indol-3-yl)-2-({[(3-methoxy-3-oxopropyl)sulfanyl]carbonothioyl}amino)propanoate* (**6d**): 1H-NMR (400.1 MHz, CDCl_3_): 8.18 (s, 1H), 7.52–7.09 (m, 4H), 6.97 (s, 1H), 5.47–5.54 (m, 1H), 4.10 (q, 2H), 3.70 (s, 3H), 3.50–3.54 (m, 1H), 3.38–3.45 (m, 2H), 3.21–3.34 (m, 1H), 2.63–2.85 (m, 2H), 1.11 (t, 3H). ESI-MS in positive mode *m*/*z*: [M − H]^+^: 395.05. [α]${}_{D}^{25}$ = +13.4 ± 1.06 (*c* 0.1, MeOH).

*Methyl 3-(1H-indol-3-yl)-2-({[(2-methyl-4-oxopentan-3-yl)sulfanyl]carbonothioyl}amino)propanoate* (**6e**): 1H-NMR (400.1 MHz, CDCl_3_): 8.19 (s, 1H), 7.60–7.15 (m, 4H), 7.13 (s, 1H), 4.4–4.60 (m, 1H), 3.75 (s, 3H), 3.36–3.49 (m, 1H), 3.34–3.40 (m, 1H), 2.17 (s, 2H), 1.63 (s, 2H), 1.25 (s, 3H), 1.11 (s, 3H). ESI-MS in positive mode *m*/*z*: [M − H]^+^: 393.35. [α]${}_{D}^{25}$ = +22.9 ± 0.611 (*c* 0.1, MeOH).

*Ethyl 3-(1H-indol-3-yl)-2-({[(2-methyl-4-oxopentan-3-yl)sulfanyl]carbonothioyl}amino)propanoate* (**6f**): 1H-NMR (400.1 MHz, CDCl_3_): 8.21 (s, 1H), 7.60–7.20 (m, 4H), 7.15 (s, 1H), 4.48–4.56 (m, 1H), 4.21 (q, 2H), 3.50–3.54 (m, 1H), 3.34–3.40 (m, 1H), 2.15 (s, 2H), 2.01 (s, 3H), 1.37 (s, 3H), 1.28 (t, 3H), 1.11 (s, 3H). ESI-MS in positive mode *m*/*z*: [M − H]^+^: 407.15. [α]${}_{D}^{25}$ = −87.5 ± 0.643 (*c* 0.1, MeOH).

### 3.6. Antifungal Assay

Compounds **4**–**7d** were in vitro evaluated for their antifungal activity against *F. oxysporum* using an amended-medium protocol to assess the mycelial growth inhibition. Potato dextrose agar (PDA) medium was then prepared in flasks and sterilized. Compounds **4**–**7d** were vigorously mixed with PDA in order to get a final concentration of the test compounds in the medium in a 10–300 μg/mL range. Amended-medium was then poured into sterilized 24-well plates. An isolate of *F. oxysporum* (G1) was incubated in PDA at (20 ± 1) °C for 8 days to get new mycelium for the antifungal assays. 2-mm-diameter mycelial plugs were thus inoculated in the center of each PDA-containing well. The inoculated 24-well plates were incubated at (20 ± 1) °C for 5 days. PDA (neat) was used as control. Three replicates were performed for each treatment. The radial mycelial growth of the fungal colonies was measured in comparison to that of the control, using the ImageJ software. The mycelial growth inhibition of the test compounds was calculated via the formula: inhibition growth (%) = (C − T) × (100/C), where C represents the diameter of fungal growth on untreated PDA, and T represents the diameter of fungal growth on treated PDA. Inhibition growth (%) data were then used to build the corresponding dose-response curves in order to calculate the half-maximal inhibitory concentrations (IC_50_ expressed in mM) for each compound, using a non-linear regression in the program GraphPad Prism version 5.00 (GraphPad Software, San Diego, CA, USA) for Windows.

## 4. Conclusions

In summary, we have synthesized novel indole analogs derived from l-tryptophan, identifying the main reaction conditions for the different mechanistic routes towards formation of **4**, **5**, or **6**. In addition, an in vitro antifungal testing against *Fusarium oxysporum* was performed for all compounds, demonstrating the promissory behavior of the synthesized compounds as templates for the development of a novel kind of antifungal agents.

## Figures and Tables

**Table 1:** Antifungal activity of the compounds **4**–**7** against *Fusarium oxysporum*.

Compound	IC_50_^a^ (mM) ± SD
**4a**	0.49 ± 0.09
**4b**	0.76 ± 0.23
**4c**	1.5 ± 0.5
**4d**	1.1 ± 0.2
**5**	1.8 ± 0.9
**6a**	2.5 ± 0.7
**6b**	0.16 ± 0.05
**6c**	2.1 ± 0.8
**6d**	1.1 ± 0.7
**6e**	0.59 ± 0.21
**6f**	1.7 ± 0.5
**7a**	3.1 ± 1.7
**7b**	>50
**7c**	7.9 ± 3.8
**7d**	0.76 ± 0.14

^a^ Data expressed as mean values ± standard deviation (SD) of three replicates.
